# Comparison between the Airgo™ Device and a Metabolic Cart during Rest and Exercise †

**DOI:** 10.3390/s20143943

**Published:** 2020-07-15

**Authors:** Andrea Antonelli, Dario Guilizzoni, Alessandra Angelucci, Giulio Melloni, Federico Mazza, Alessia Stanzi, Massimiliano Venturino, David Kuller, Andrea Aliverti

**Affiliations:** 1 Allergologia e Fisiopatologia respiratoria, Azienda Ospedaliera Santa Croce e Carle, 12100 Cuneo, Italy; antonelli.a@ospedale.cuneo.it; 2 Dipartimento di Elettronica, Informazione e Bioingegneria, Politecnico di Milano, 20133 Milan, Italy; dario.guilizzoni@gmail.com (D.G.); andrea.aliverti@polimi.it (A.A.); 3 Chirurgia Toracica, Azienda Ospedaliera Santa Croce e Carle, 12100 Cuneo, Italy; melloni.g@ospedale.cuneo.it (G.M.); mazza.f@ospedale.cuneo.it (F.M.); alessia.stanzi@gmail.com (A.S.); venturino.m@ospedale.cuneo.it (M.V.); 4 MYAIR Inc., Boston, MA 02116, USA; david.kuller@gmail.com

**Keywords:** wearable devices, respiratory monitor, respiratory function, respiratory Holter

## Abstract

The aim of this study is to compare the accuracy of Airgo™, a non-invasive wearable device that records breath, with respect to a gold standard. In 21 healthy subjects (10 males, 11 females), four parameters were recorded for four min at rest and in different positions simultaneously by Airgo™ and SensorMedics 2900 metabolic cart. Then, a cardio-pulmonary exercise test was performed using the Erg 800S cycle ergometer in order to test Airgo™’s accuracy during physical effort. The results reveal that the relative error median percentage of respiratory rate was of 0% for all positions at rest and for different exercise intensities, with interquartile ranges between 3.5 (standing position) and 22.4 (low-intensity exercise) breaths per minute. During exercise, normalized amplitude and ventilation relative error medians highlighted the presence of an error proportional to the volume to be estimated. For increasing intensity levels of exercise, Airgo™’s estimate tended to underestimate the values of the gold standard instrument. In conclusion, the Airgo™ device provides good accuracy and precision in the estimate of respiratory rate (especially at rest), an acceptable estimate of tidal volume and minute ventilation at rest and an underestimation for increasing volumes.

## 1. Introduction

Respiratory rate measurement has been shown to be able to predict adverse clinical events, such as admission to the Intensive Care Unit (ICU). Under specific circumstances, it is more effective than pulse or blood measurements at discriminating between stable patients and patients at risk [1,2]. However, the number of trials in which the respiratory rate has been studied remains limited and mostly confined to the use of spot measurements.

The work by Yañez et al. [3] is based on a direct measurement of the flow to assess the respiratory frequency in COPD patients. In this study, it was observed than the mean respiratory rate was raised 15 days prior to hospitalization (two days before, 15% more with respect to the baseline; the day before, 30% more with respect to the baseline). The work by Shah et al. [4] concludes that the respiratory rate, in that case estimated by a probe pulse oximeter, is predictive of a COPD exacerbation: mean respiratory rate increased from 22 to 24 breaths per minute. Furthermore, it was reported in the literature that respiratory rate can predict serious cardiac illnesses such as cardiac [2] and cardiopulmonary [1] arrest and heart failure [5]. Finally, a recent study on the novel COVID-19 pointed out that respiratory rate is one of the most predictive signals of worsening of patients’ conditions [6].

A limitation that is found in several studies, like the previously mentioned ones, is that physiological parameters were measured with spot measurements and when the subject is at rest, while it is known that physical activity has an influence on cardiorespiratory function [7].

Therefore, the continuous assessment of respiratory function outside of laboratory settings and during different levels of activity can provide a tool for the early detection of worsening chronic respiratory conditions (exacerbations) [7] or monitoring patient deterioration in acute diseases such as COVID-19 [8], thus improving the quality of life of the patients, reducing mortality, and limiting the costs sustained by healthcare systems.

Protocols for the continuous monitoring of breathing can use either wearables (e.g., respiratory inductance plethysmography [9], resistance-based sensors or garments [10,11], capacitance-based sensors [12], inertial measurement units [13,14], fiber optic sensors [15], acoustic sensors [16], or a combination of more techniques [17]) or non-wearable devices (e.g., contactless methods [18]), however this last category is constituted by more cumbersome methods that are uncomfortable to use in daily-life settings [19].

An effective way to continuously assess respiratory function for long periods is through the integration of wearable respiratory monitors in telemedicine platforms, which collect, store and process data and provide a feedback for the clinicians [20]. Furthermore, professional and amateur athletes could use these devices to track trends and improvements in their performances and physiological parameters; in fact, respiratory frequency is a good marker of physical effort and is closely associated with perceived exertion [21].

## 2. Materials and Methods

The goal of the presented work is to compare a metabolic cart, considered a gold standard, with Airgo™ (MYAIR Inc, Boston, MA, USA; MyAirgo Italy Srl, Milan, Italy), a resistance-based wearable device able to derive breathing parameters from body surface motion detection acquired at the level of the lower ribcage. A study protocol has been conducted on a population of twenty-one healthy subjects comparing the output of the Airgo™ system with the SensorMedics 2900 metabolic cart (SensorMedics Inc., Yorba Linda, CA, USA) [22]. The research has been approved by the Ethical Committee of the Azienda Ospedaliera S. Croce e Carle in Cuneo (Opinion number 3–17, 7 June 2017) and was part of the clinical trial NCT03368612 (“Comparative study between the Airgo™ system and standard tests in the assessment of the respiratory function”).

In the first part of the protocol, the healthy subjects were asked to perform quiet breath maneuvers in five different standardized postures while wearing the Airgo™ device: standing position, sitting position with back against chair, supine position, right lateral decubitus and left lateral decubitus.

In the second part of the test, after the completion of the test at rest, a symptom-limited incremental exercise was performed on the electronically braked cycle ergometer Ergoline 800s (ergoline GmbH, Bitz, Germany).

The obtained data have been processed to allow the extraction of relevant respiratory parameters. The comparison between the two systems was done considering the following four parameters: normalized tidal volume, respiratory rate, normalized minute ventilation and duty cycle.

### 2.1. Description of the Hardware

**Figure 1:**
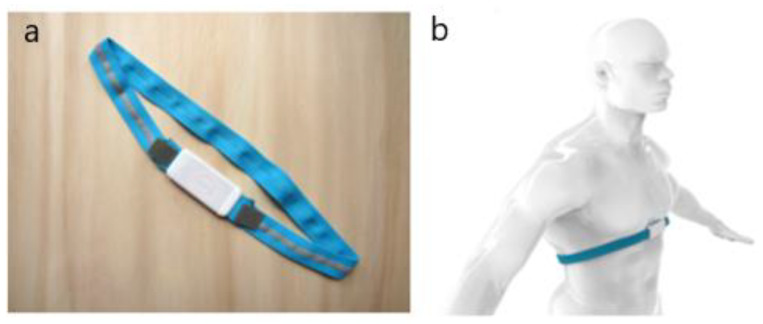
(**a**) The Airgo™ band; (**b**) Correct positioning of Airgo™ around the subject’s torso.

The Airgo™ band measures the thoracic circumference changes with a stretchable knitted matrix of nylon and spandex with a knitted-in silver coated yarn. The system employs an electrically conductive, elastomeric chest band that encircles the lower chest and floating ribs of a subject. In Figure 1, the Airgo™ band and an example of the correct fitting are reported.

The system calculates resistance changes continuously. Each variation in resistance values, caused by each expansion and volume reduction of the chest wall during breathing activity, reflects a measurable variation in current in the silver-coated wire.

The stretchable band is coupled to a microprocessor, embedded in a sintered nylon shell. The microprocessor includes an SD memory card and monitors the respiratory activity by collecting raw data at a frequency of 10 Hz. The microprocessor includes: an analog-to-digital converter (ADC) that converts the analog resistance level of the girth band into a 10-bit number ranging from 0 to 1023 and corresponding to the amplitude of the torso expressed in arbitrary units; a battery for operating the microprocessor and for charging the band; paths to transfer data to the SD card for on-board data storage; a Bluetooth module to wirelessly communicate with a computational device (laptop computer or smartphone). The device’s on-board microprocessor is connected to a nine-axis inertial measurement unit (IMU). The motion detection circuit provides movement and postural orientation datapoints with the final aim to associate breathing information to the patient’s posture or activity. An activity recognition algorithm based on raw accelerometer data has been developed and is described by Qi and Aliverti [23].

The girth band is operably coupled at the first end to the microprocessor and then encircles the torso at the level of the lower rib cage. It is then coupled at the second end to the microprocessor from the other side. When in use, the band is pre-tensioned to ensure a fit around the torso that does not dislocate when the band is worn and can record data without interruption.

The part in direct contact with the skin is made of a silicon rubber with a thickness of about 0.35 mm called GECKO^®^ (Gottlieb Binder GmbH & Co. KG, Holzgerlingen, Germany), or gecko tape, because it emulates the adhesion of gecko feet and can be attached to both wet and dry vertical surfaces. This material can be detached easily and quickly from a surface, while it shows exceptional adhesion properties when attached to it.

**Figure 2:**
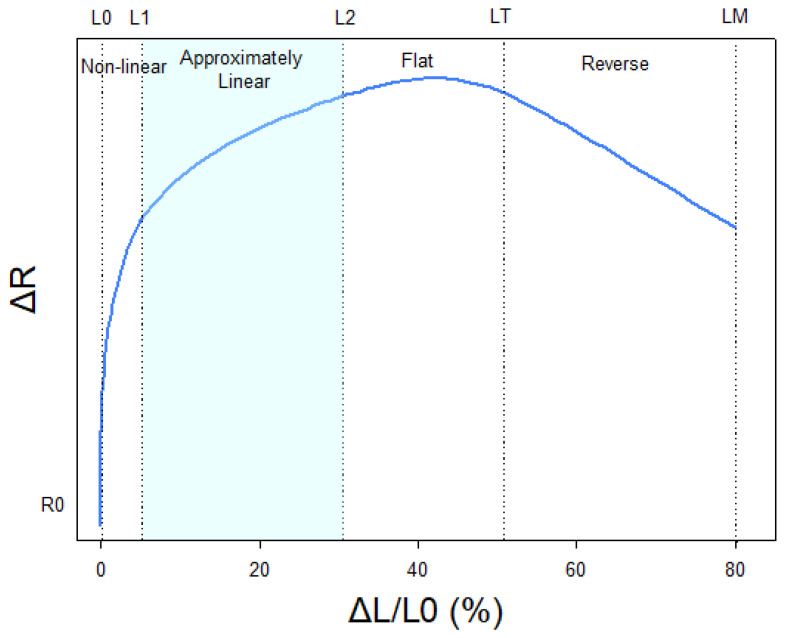
Stretch metallic knit “static” resistance qualitative curve. The graph shows the non-linear resistance/stretch curve of the resistive fabric and the different ranges of resistance (non-linear, approximately linear, flat and reverse, with LT the length threshold above which resistance decreases and LM indicating the maximum length). The section in evidence indicates the operative range.

Figure 2 shows a typical stretch metallic knit static resistance qualitative curve. Initially, the resistance rapidly changes with elongation (L0 to L1). Following this initial phase, there is an approximately linear increase in resistance with respect to length (L1 to L2). Resistance continues to increase with length but with less marginal increment, as shown from L2 to LT, which is the threshold above which resistance decreases with increasing length until the elastic band reaches its maximum length (LM). The operative range of the band is therefore within the range L1–L2 in order to obtain accurate measurements without significant calibration difficulties.

### 2.2. Study Design

This study is a monocentric, prospective, observational, single arm clinical trial involving 21 healthy volunteers. It was conducted at the Azienda Sanitaria Ospedaliera (ASO) S. Croce e Carle, Allergologia e Fisiopatologia Respiratoria, Cuneo (CN), Piemonte. The 21 healthy subjects comprised 11 females and 10 males with a mean age of 36 (age range from 24 to 51), mean weight of 67 kg (standard deviation ±16 kg) and mean height of 170 cm (standard deviation ± 8 cm).

In order to be enrolled in the study, the following inclusion criteria have been used:

- Non-smoking healthy volunteers;
- Volunteers able to perform a routine respiratory function test (spirometry and cardiopulmonary exercise testing);
- Age between 18 and 75;
- Volunteers able to understand an informed consensus.

The exclusion criteria have been the following:

- Presence of cardiac comorbidities (e.g., coronary artery diseases, heart failure, arrhythmias);
- History of coronary artery disease of heart failure;
- Presence of respiratory comorbidities (e.g., asthma, COPD, sleep apnea, lung interstitial disease);
- Pregnancy;
- Former smoker of >5 pack-year;
- Severe trauma or major surgery in the last year;
- Chest pain;
- Obesity (BMI > 30).

### 2.3. Acquisition Protocol

The acquisition protocol was divided into three main phases:

(1) *Preparation:* measurement of the subject’s thoracic oblique circumference length (C) at the end of a forced expiration maneuver. Knowing this measurement, the initial length of the elastic band (L0) was decided to be 7% shorter than C in order to obtain a pre-tensioned girth band to ensure an effective fit around the torso. After being cut, the band was coupled with the Airgo™ electronic device and positioned against the subject’s skin;
(2) *Test at rest*: recording of respiratory parameters while letting the subject breathe quietly in five different standardized positions (standing, seated, supine, right lateral decubitus, left lateral decubitus) for 4 min. The supine position required an elevation of the subject’s head not greater than 10° with a pillow under the subject’s head. Tidal volume, respiratory rate, minute ventilation, inspiratory time, expiratory time and duty cycle (explained in Table 1) were simultaneously recorded by the Airgo™ system and the SensorMedics 2900 metabolic cart. In order to facilitate the off-line synchronization between the two systems, subjects were asked to perform a big, deep breath at the beginning of the test for each position;
(3) *Test under physical exercise:* execution of a cardiopulmonary exercise test on the Ergoline cycle ergometer 800S, followed by a recovery period. A symptom-limited incremental exercise test was performed and designed to achieve a maximum load in 10 ± 2 min in each subject wearing the Airgo™ device. The physical exercise test followed a linear incremental protocol (with a slope between 15 and 20 Watts per minute) was identical for men and women and chosen based on the level of training of each subject so that each test lasted approximately between 6 and 12 min, as recommended in the guidelines. Subjects were asked to cycle at a velocity of 60 revolutions/minute for 2 min with no load, then to pedal at incremental workload maintaining the same speed as before until the maximal effort was reached. Once the subject reached his/her maximal effort and he/she was not able to ride anymore, the exercise test was interrupted, and the recovery phase started. This phase consisted of cycling for 2 min without any resistance followed by 2 min at rest. The same respiratory parameters as test at rest have been acquired with the big-breath synchronization maneuver.

### 2.4. Airgo™ Signal Processing Algorithm

Airgo™’s processing unit acquires a respiratory signal based on the change in girth band resistance over time. Many other factors not related to the breath cycle may influence the stretched length of the band, causing girth measurement inflections at a much higher frequency than that of the breath. Therefore, raw data have been processed to filter out motion and heartbeat artefacts. The latter are characterized by a smaller amplitude and a higher frequency with respect to the respiratory signal. The system samples data at a frequency of 10 Hz, averages the acquired data over 9–34 reading, blurs the averaged data from 0.3 to 1 s to filter out artefacts, determines the beginning and the end of a breath and records an adverse event if a predetermined period of time has elapsed without detecting a new breath, as is described in the related patent (number US201462007142P).

**Figure 3:**
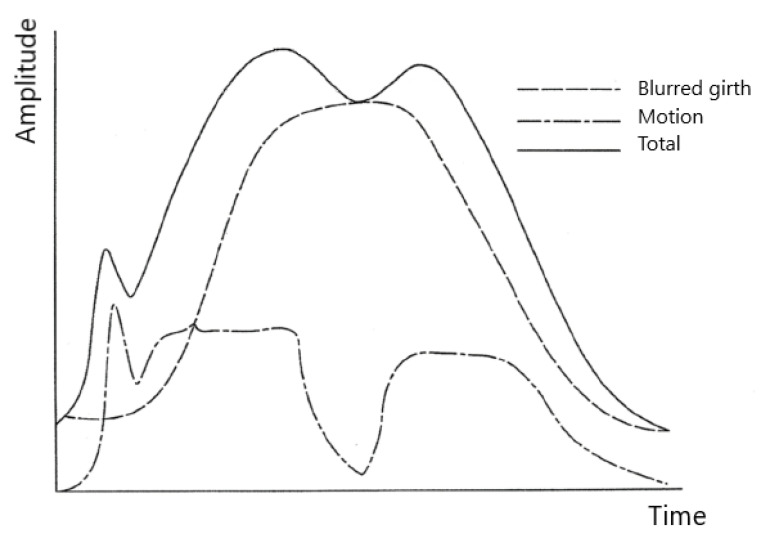
Influence of knit motion and acceleration artefacts (blurred girth) on the breath signal read by the Airgo™ band (modified from patent number US201462007142P).

Filtering has an effect on knit motion artefacts, too. The total measured breath cycle resembled an upside-down “W” (Figure 3): this non-correspondence is not only due to the heartbeat but also to the motion and acceleration artefacts of the signal coming from the knitted metallic band. A portion of the resistance is static and related to the static length of the stretched knit (the overall bell shape), but part of the resistance is also due to the spontaneous motion of the fabric. This accounts for the slight hump at the beginning of the total measured breath cycle and the double hump in the middle.

**Figure 4:**
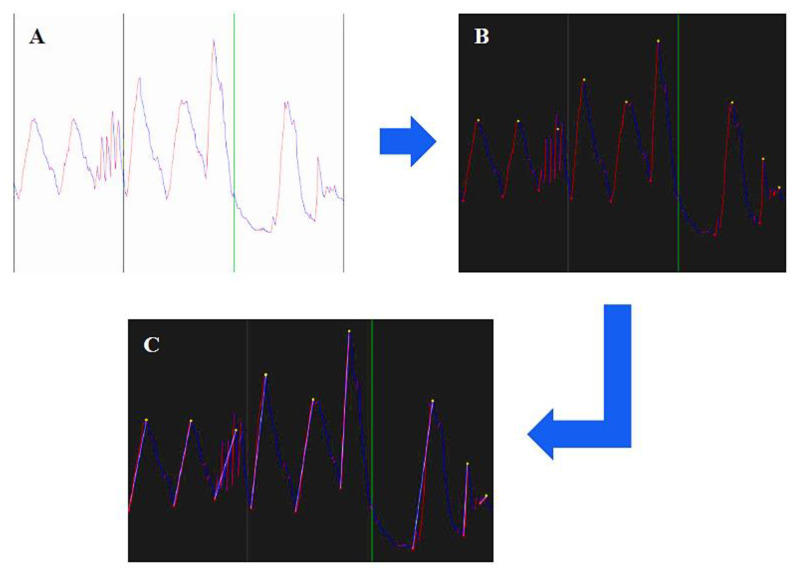
Raw data extraction process. (**A**) 30 s of raw data (red during inhalation, blue during exhalation; (**B**) absolute maximum/minimum points found for each cycle; (**C**) vector representation of the inhalation phase.

Once raw data are filtered, they are processed in order to obtain the proper breath signal. The main steps of this process, shown in Figure 4, can be summarized as follows:

- Identification of maximum and minimum of each breathing cycle;
- Representation of each breath by means of a vector that connects the maximum and the minimum;
- Automatic reconstruction of segmented breath, e.g., due to obstructions or movement;
- Automatic removal of fake breaths;
- Computation of tidal volume, respiratory rate and minute ventilation.

Breath data can be then transmitted to a computer via Bluetooth Low Energy, stored, visualized and further analyzed using Airgo™’s dedicated software.

### 2.5. Features Extraction

**Figure 5:**
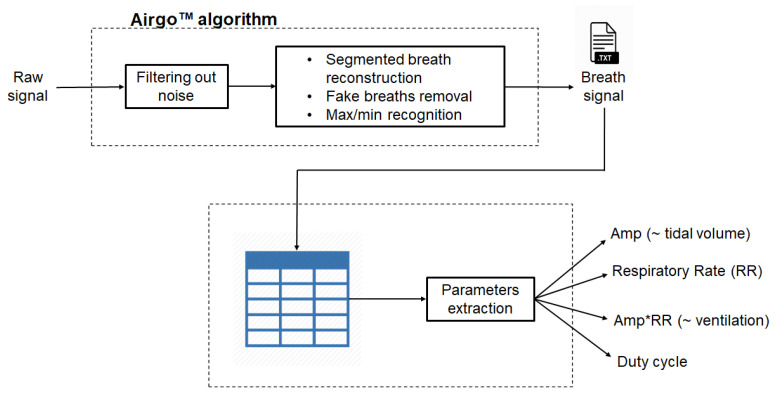
Box scheme of the raw signal processing and extraction process of the parameters of interest: amplitude (Amp), which represents Airgo™’s signal variation and models SensorMedics’ tidal volume; respiratory rate (RR); Airgo™’s minute ventilation (Amp×RR), which models SensorMedics’ minute ventilation; duty cycle, which is the ratio between inspiratory time and total breath period.

The signal acquired by the girth band is expressed in arbitrary units ranging from 0 to 1023 (10-bit analog to digital converter) and expresses the amplitude of the signal recorded in the range of differential potential levels between 0.5 and 3.6 V, which is the microprocessor supply voltage. This signal represents the signal amplitude variation due to the trunk expansion and reduction during breathing. In Figure 5, an explicative diagram of the feature extraction process is reported.

As the breath period (T) is computed as the temporal distance between two consecutive minima, the respiratory rate is the inverse of the breath period expressed as breaths per minute and the expiratory time is computed as the difference between breath period and the inspiratory time.

Among SensorMedics’ data, outliers, fake breaths and missing data were detected and deleted. These errors may be due to the incorrect positioning of the mouthpiece and the consequent air leakage. In these cases, it is necessary not to consider the entire breath.

### 2.6. Alignment and Data Organization

In order to validate the Airgo™ system, a breath-by-breath comparison with the SensorMedics outputs was performed. Since the amplitude (Amp) represents the same features as the tidal volume read by the metabolic cart and expressed in liters, the values obtained with the two devices had to be normalized in order to be compared. For each position of each subject, the mean of the first twenty values of quiet breathing after the initial big breath was computed and used as a reference. This leads to normalized values for Airgo™’s amplitude and minute ventilation and the corresponding SensorMedics’ volume and minute ventilation in all conditions, allowing to derive four relative parameters:

- Airgo™’s normalized amplitude (Amp_A_/Amp_A0_);
- Airgo™’s normalized minute ventilation (MV_A_/MV_A0_);
- SensorMedics’ normalized tidal volume (Vt_SM_/Vt_SM0_);
- SensorMedics’ normalized minute ventilation (Vt_SM_/Vt_SM0_).

The alignment of the two signals covered a fundamental role. In order to facilitate the process of identification and extraction of the correct signal window referring to a given position to be compared in the two instruments, subjects have been asked to perform a deep, big breath at the beginning of each test in each position. The comparison between the breath signals recorded by the Airgo™ device and the SensorMedics metabolic cart is based on the number of breaths in the same period and on the big-breath maneuver correspondence. After this phase, in order to obtain the correct number of breaths in the same time span, all Airgo™ values representing heartbeat artefacts or fake breaths have been filtered out according to the Airgo™ signal processing algorithm explained in Section 2.4.

With the Airgo™ software it was possible to reconstruct two segmented contiguous breaths that are recognized activities by linking the minimum of the first breath to the maximum of the second.

**Figure 6:**
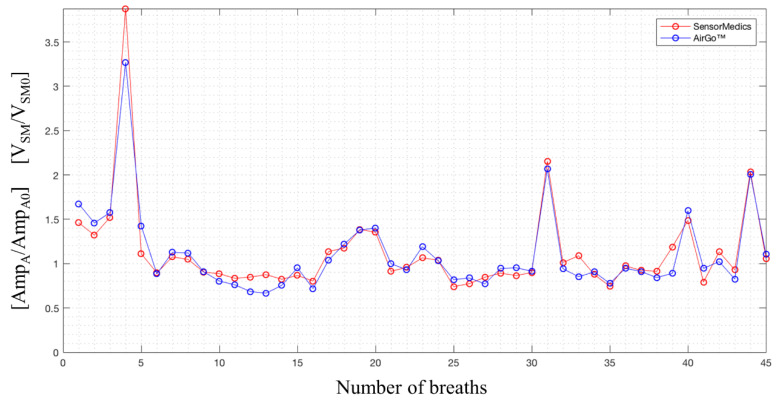
SensorMedics’ normalized volumes and Airgo™’s normalized amplitudes synchronized, standing position, healthy female subject (38 years old, 165 cm, 52 kg).

**Figure 7:**
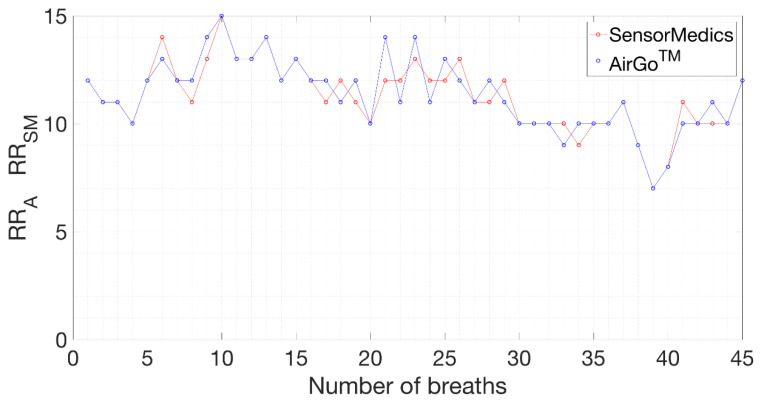
SensorMedics’ and Airgo™’s respiratory rates synchronized, standing position, healthy female subject (38 years old, 165 cm, 52 kg).

Thanks to the synchronization with the big breaths, the same number of breaths as in SensorMedics file in the same period was determined. However, when performing the alignment, a progressive alignment shift between the two measurements after the big breath was noticed. This was due to a temporal delay in the Airgo™ system’s sampling process that led to a delay of about 3–4 s at the end of each position test. To overcome this problem and visualized the two sequences of values correctly, it was necessary to represent the outputs of Airgo™ and SensorMedics with respect to the number of breaths of each position test instead of the breath period. An example of synchronization of the amplitude outputs in a static position is shown in Figure 6, while an example of synchronization of the respiratory rate outputs is shown in Figure 7.

**Figure 8:**
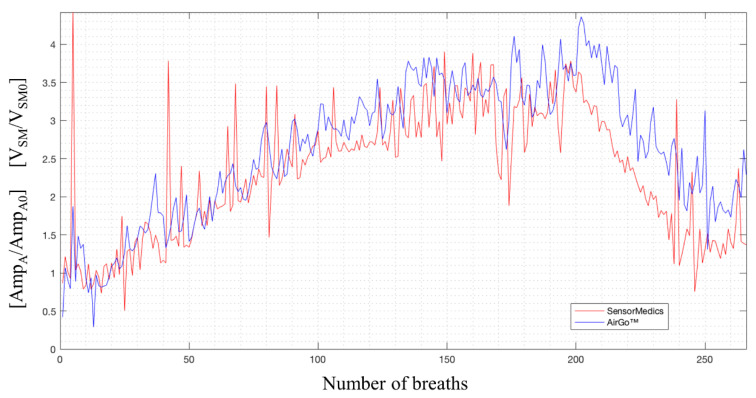
SensorMedics’ normalized volumes (in red) and Airgo™’s normalized amplitudes (in blue) synchronized, exercise test, healthy male subject (45 years old, 169 cm, 62 kg).

In exercise test recordings, because of movement artefacts, noises coming from changes in posture, air losses through the mouthpiece caused by the increasing level of physical effort and the greater amount of breaths than the test at rest, the alignment process was based only on the initial big breath synchronization, so the final shift in alignment was not eliminated. An example is reported in Figure 8, where another common problem is shown: for volumes higher than the tidal volume, Airgo™’s normalized amplitudes tend to underestimate the SensorMedics’ normalized volume.

In order to improve the effectiveness of the statistical analysis and to allow comparisons between subjects with different levels of maximum workload, the entire exercise test was divided in four different regions according to the increasing physical stress intensity: low intensity (“L”), medium intensity (“M”), high/maximum intensity (“H” and “I_MAX_”) and recovery phase (“RP”). To identify the maximum of exercise intensity for each subject, the SensorMedics’ maximum value of normalized value of minute ventilation was considered as reference.

To equally divide the four regions, two points of reference were identified, one at one third and the second at two third of the normalized minute ventilation maximum value of the subject. Within the first three regions characterized by an increasing ventilation, four smaller sections constituted by at least twenty breaths were identified: the first one referring to the low intensity region (“L”), the second one referring to the medium intensity region (“M”), while the third and fourth referring to the high and maximum intensity region (“H” and “IMAX”).

**Figure 9:**
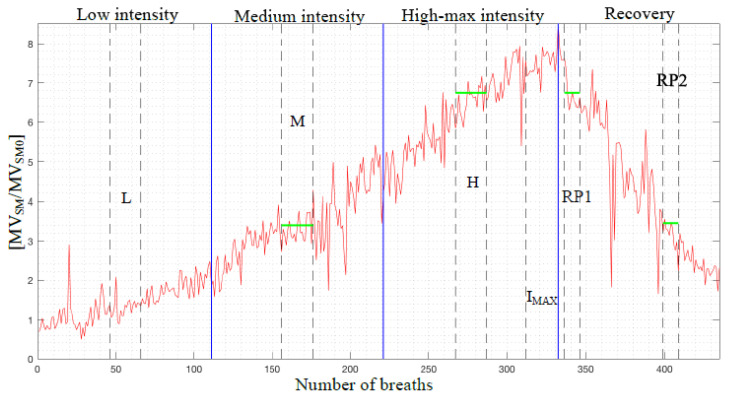
Normalized minute ventilation signal and regions identified, exercise test and recovery (healthy female subject, 38 years old, 165 cm, 52 kg). The green segments indicate the correspondence of normalized minute ventilation values between RP1 and H, and RP2 and M sections, respectively.

Because each subject reached a different maximum load according to his/her physical capacity, the four sections were centered differently from subject to subject. The section “L” was constituted by the central breaths of the first region, the section “M” by the central breaths of the second region; the section “H” by the central breaths of the third region and the section “I_MAX_” by the last twenty breaths of the exercise text track before the section “RP”. In this last region, two other smaller sections were identified: the first one (“RP1”) containing at least ten breaths centered around the closest normalized minute ventilation central value within the “H” section and the second one (“RP2”) containing at least ten breaths centered around the closest ventilation central value within the “M” section. An example of this division into sections in the case of the minute ventilation can be seen in Figure 9.

### 2.7. Statistical Analysis

In order to verify the goodness-of-fit of values distribution, the Kolmogorov–Smirnov test of normality has been run both on single distributions and on the differences between the two methods. Since the test returned that data did not come from a normal distribution, a non-parametric statistical analysis has been conducted.

To have a general overview of Airgo™’s estimate error in each position both during quiet breath and during the exercise test for each subject, relative error medians, interquartile ranges (IQRs) and limits of agreement (LOAs) were calculated for each parameter. Then, to find a significant difference between different positions and intensity levels (within the exercise test), the One-Way Kruskal–Wallis test has been conducted with a level of significance of *p* < 0.05.

## 3. Results

In the next paragraphs, box and whiskers plots of the differences between the two methods are reported for each position and for the two conditions (static postures and exercise test). An example of a Bland–Altman plot for a subject is reported to graphically show the agreement between the two instruments.

### 3.1. Relative Error Medians, Interquartile Ranges and Limits of Agreement of the Parameters

The relative error median percentage, the interquartile range and the limits of agreement of the parameters during the test at rest and the exercise test are reported in Table 2 and Table 3, respectively. Negative signs in the percentages stand for an understimation of the Airgo™’s computed parameters with respect to those of SensorMedics 2900, and negative values indicate the amount of the understimation.

In the case of the respiratory rate, the overall relative error median percentage in all positions was of 0%, with the highest value of interquartile range of 15.0 in the supine position.

The second best result was the overall relative error median percentage in the case of the normalized amplitude parameter: in fact, in the supine position the percentage of 0.8% was the lowest relative error for the test at rest after the results of the respiratory rate. Moreover, by looking at changes in the relative error median between positions for the normalized amplitude parameter it was possible to notice a difference in values between standing and horizontal positions (supine, right lateral decubitus, left lateral decubitus). In particular, for horizontal positions the overall percentage values were lower (0.8%, 4.4% and 1.1%) compared to the seated position (9.0%) with the standing position percentage value standing between the two (6.8%).

The normalized minute ventilation parameter, calculated as the product of tidal volume and respiratory rate, gained similar results to the normalized amplitude parameter and it was affected by similar errors in the same positions.

To obtain representative error values for each respiratory parameter independently from the position, considering the mean error in all positions, the relative errors were 4.4% for normalized amplitude, 0% for respiratory rate, 4.2% for normalized ventilation and −0.3 for duty cycle.

As shown in Figure 2, Airgo™’s estimates tend to underestimate with increasing volumes and consequently with increasing ventilation during exercise, as was graphically shown in Figure 8. In the normalized amplitude values reported in Table 3, the relative error increased drastically from −11.1% at low intensity to −39.7% at maximum intensity. In the second recovery phase, the overall median relative error was −24.8%, a value that stands in between the low-intensity and medium-intensity values. This result was also more evident by looking at the overall normalized ventilation relative error medians.

In this case, the respiratory rate was confirmed as the best estimate with an overall relative error median percentage of 0% in all physical effort conditions, while the duty cycle parameter had the highest error of −0.27 in the medium intensity level of the exercise.

### 3.2. Kruskal–Wallis Tests between Positions and Intensities

The results of One Way Kruskal–Wallis analysis are reported in Table 4 for the test at rest in different positions and for the exercise test at different intensity levels.

For the test at rest, normalized amplitude, normalized ventilation and respiratory rate parameters were independent on position (*p* > 0.05). On the other hand, duty cycle was influenced by posture, with a statistically significant difference between standing and supine positions (*p* < 0.05).

For the exercise test, the respiratory rate was the only parameter which resulted to be independent from different levels of exercise intensity (*p* > 0.05). In the cases of normalized amplitude (*p* < 0.01), normalized ventilation (*p* = 0.001) and duty cycle (*p* < 0.01) parameters, there was a significant effect of the intensity level of exercise on the error. In particular, the post hoc pairwise comparison highlighted that the first two parameters presented a statistically significant difference between conditions L and H (*p* < 0.05 for normalized amplitude and *p* < 0.01 for normalized ventilation), and conditions L and I_MAX_ (*p* = 0.014 for normalized amplitude and *p* < 0.01 for normalized ventilation). The duty cycle presented, instead, a significant difference between M and RP1 conditions (*p* < 0.01), and M and RP2 conditions (*p* < 0.05).

### 3.3. Box and Whiskers Plots

**Figure 10:**
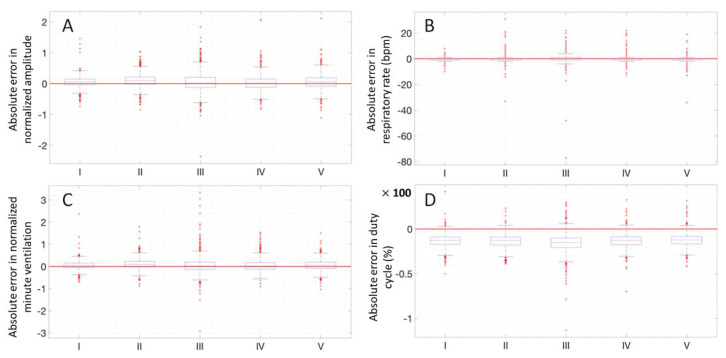
Absolute error box plots, all subjects, different positions at rest, all parameters: normalized amplitude (**A**), respiratory rate (**B**), normalized minute ventilation (**C**) and duty cycle (**D**). The positions shown are the following: standing (I), seated (II), supine (III), right side (IV), left side (V).

**Figure 11:**
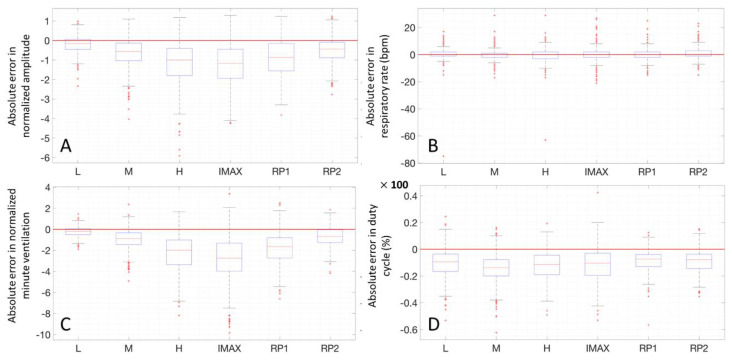
Absolute error box plots, all subjects, different intensities during exercise, all parameters: normalized amplitude (**A**), respiratory rate (**B**), normalized minute ventilation (**C**) and duty cycle (**D**). The intensities shown are the following: low (L), medium (M), high (H), maximum (IMAX), recovery phase 1 (RP1), recovery phase 2 (RP2).

In Figure 10, the four parameters in the different positions for all subjects are reported in boxplots, where the different positions of the test at rest are indicated with Roman numbers. The same analysis has been performed on the parameters during the exercise test and the result is shown in Figure 11.

### 3.4. Bland–Altman Plots

**Figure 12:**
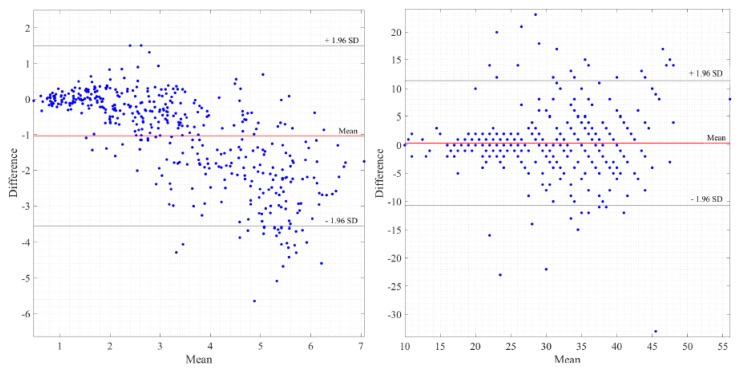
Bland-Altman plots, exercise and recovery test, normalized ventilation (**left**) and respiratory rate (**right**) in a healthy female subject (38 years old, 166 cm, 52 kg).

The Bland–Altman plot allows us to appreciate how much the system being validated differs from the reference system. The results obtained in the case of a healthy female subject (38 years old, 166 cm, 52 kg; same subject shown in Figure 6, Figure 7 and Figure 9) are reported in Figure 12 in the case of exercise and recovery tests for normalized ventilation and respiratory rate parameters. The obtained results are not significantly different from the results obtained in the previously illustrated representation methods. Focusing on normalized ventilation (Figure 12, left), points tended to decrease with increasing ventilation, thus displaying the behavior of a proportional error. Respiratory rate (Figure 12, right) showed a constant variability of the error.

**Figure 13:**
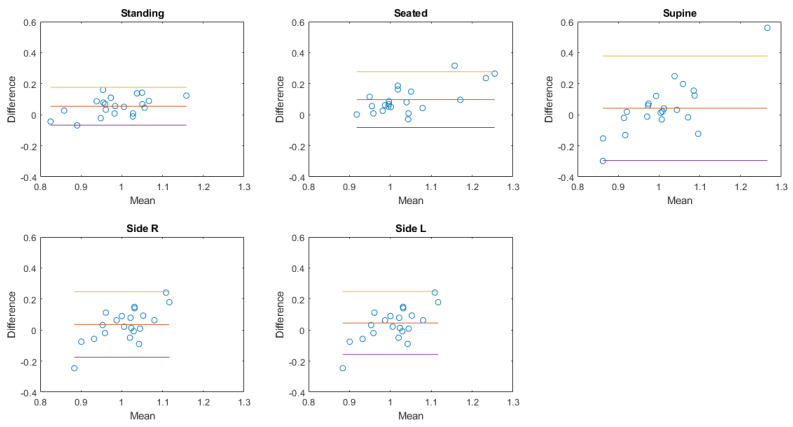
Bland-Altman plots of the aggregated results of normalized amplitude at rest. Each dot represents the value obtained from a single subject. The yellow line represents the upper limit of agreement (mean error +1.96× standard deviation); the red line represents the mean error; the purple line represents the lower limit of agreement (mean error −1.96× standard deviation).

**Figure 14:**
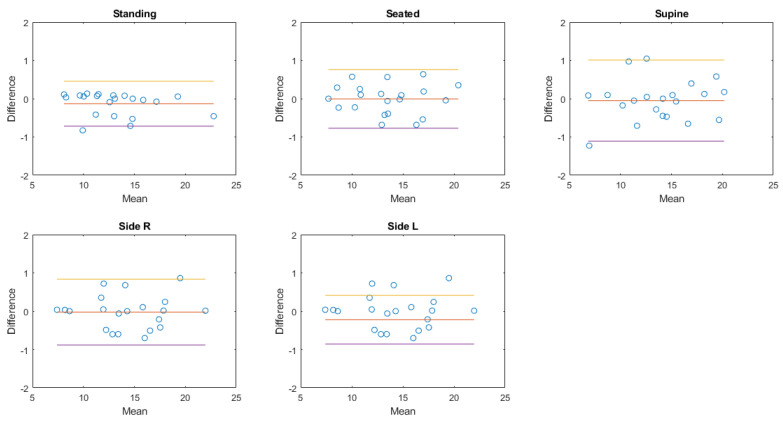
Bland–Altman plots of the aggregated results of respiratory rate at rest. Each dot represents the value obtained from a single subject. The yellow line represents the upper limit of agreement (mean error +1.96× standard deviation); the red line represents the mean error; the purple line represents the lower limit of agreement (mean error −1.96× standard deviation).

**Figure 15:**
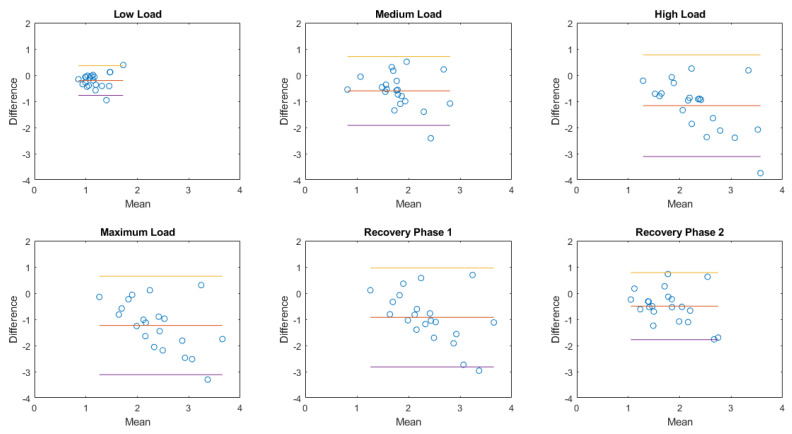
Bland–Altman plots of the aggregated results of normalized amplitude during exercise. Each dot represents the value obtained from a single subject. The yellow line represents the upper limit of agreement (mean error +1.96×standard deviation); the red line represents the mean error; the purple line represents the lower limit of agreement (mean error −1.96×standard deviation).

**Figure 16:**
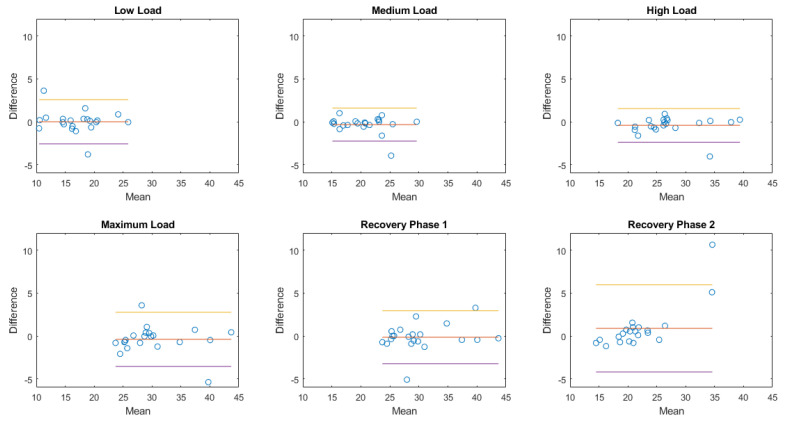
Bland–Altman plots of the aggregated results of respiratory rate during exercise. Each dot represents the value obtained from a single subject. The yellow line represents the upper limit of agreement (mean error +1.96× standard deviation); the red line represents the mean error; the purple line represents the lower limit of agreement (mean error −1.96× standard deviation).

The Bland–Altman plots of the aggregated results of normalized amplitude and respiratory rate in each posture or during each activity are then shown in Figure 13, Figure 14, Figure 15 and Figure 16.

## 4. Discussion

The comparison of the Airgo™ device with the metabolic cart showed that the respiratory rate was the most accurate parameter, in all positions, for both rest and exercise tests in all conditions of physical effort: medians were always positioned on the zero error line with low dispersion around this value. Normalized amplitude and normalized ventilation showed similar trends and similar error changes both in different positions and during exercise. At rest, the overall error was small and remained almost constant in different positions, with the supine position characterized by better results compared to the others, even though this aspect did not have statistical significance. On the other hand, Airgo™’s underestimation of increasing breath volumes during exercise was confirmed and it was also more evident in normalized minute ventilation. Additionally, the recovery phase was characterized by a progressive reduction in absolute errors, which tend to assume initial median values.

In the results of the exercise tests, it was previously observed that in the second recovery phase, the overall median relative error was −24.8%, a value that stands between the low-intensity and medium-intensity values. The reason for this error is still a subject of research. Several possible explanations might have contributed separately or in combination to this result, however it is not possible to understand the magnitude of the contribution of each of these single explanations. The first one regards the pre-tensioned length of the girth elastic band obtained during the preparation phase of the test protocol: by cutting the band at a length slightly shorter than the real thoracic circumference (−7%) to ensure an effective fitting, it is likely that, for high volumes, the band reached the saturation region of the stretch metallic knit resistance qualitative curve in Figure 2. In this case, the resistance changes with elongation fail to have a correct dynamics, exiting from the linear region. The second explanation is related to the different relative contributions of the abdomen and the chest wall to the breathing pattern according to the posture and the trunk position assumed during exercise. In fact, it is known from the literature that a progressively increased inclination of the trunk determines a progressive reduction in the chest wall contribution and a progressive increase in abdominal contribution to the tidal volume [24], while the Airgo™ band only detects changes in circumference at the level of the abdominal rib cage, thus losing information related to the abdominal contribution.

Finally, as can be seen in the box and whiskers plots, there was a constant underestimation of duty cycle in all positions at rest and also for all intensity levels during exercise. In particular, SensorMedics’ values were always within an interval between 0.4 and 0.6, while Airgo™’s estimates were between 0.2 and 0.4. This means that, in the first case, inspiratory and expiratory time have a proportion of about 1:1, while in Airgo™’s duty cycle estimation, this proportion is about 1:2. This explains the error underestimation and it could be due to imperfections in the Airgo™ processing algorithm. Specifically, the ending of a breath is determined by computational means when the girth is either smaller than what it was when the breath began or when the girth is smaller than the maximum girth in a given breath cycle by a certain amount, as specified in the related patent. The fact that the end of expiration is detected by means of a threshold might cause errors in the precise identification of the minimum. In fact, the duty cycle is computed as described in Table 1 and an underestimation of inspiratory time with respect to expiratory (and total) time causes an underestimation of the duty cycle as well. The overall error remains constant between the test at rest and the exercise test, suggesting that duty cycle could be very accurate in both conditions when this issue is solved.

The overall performance of Airgo™ in comparison with the SensorMedics metabolic cart was satisfactory and it must be noted that the research on this device, both from the point of view of the hardware and of the signal processing software, is still ongoing.

## 5. Conclusions

Respiratory rate is a vital sign used to monitor the progression of several illnesses, to predict adverse clinical events and to discriminate between patients at risk and stable patients. Given that Airgo™’s respiratory rate estimates had a good correspondence with the analyses performed with an instrument that can be considered the gold standard, it can be concluded that the Airgo™ device could be useful to monitor respiratory rate in a non-invasive and non-intrusive way during everyday activities and sleep. Applications of this device can be found in chronic respiratory diseases as well as acute pathologies, such as the novel COVID-19, where the respiratory rate is predictive of the worsening of the disease. Further research is needed on the estimations of tidal volume, minute ventilation and duty cycle, however the obtained results are encouraging and research works combining activity recognition and estimation of respiratory parameters are needed to assess the validity of the system in a non-controlled environment.

## 6. Patents

The Airgo™ device is partially described in the US Patent number US201462007142P, title “Breath volume monitoring system and method” by David Kuller for Myair LLC.

## Figures and Tables

**Table 1:** Definitions and measurement units of the parameters computed during the tests. In the case of tidal volume and minute ventilation, Airgo™ does not provide absolute values.

Parameter	Definition	Measurement Unit
Tidal volume	Amount of air moving into and out of the lungs during spontaneous breathing	Liters [L]
Respiratory rate	Number of breaths performed in a minute	Breaths/minute [bpm]
Minute ventilation	Tidal volume multiplied by respiratory rate	Liters/minute [L/min]
Inspiratory time	Duration of the inspiration phase	Seconds [s]
Expiratory time	Duration of the expiration phase	Seconds [s]
Duty cycle	Inspiratory time divided by total time	Percentage [%]

**Table 2:** Test at rest, all positions, all parameters. Aggregated data are expressed as relative error median percentage, interquartile range (IQR) and limits of agreement (LOAs), computed as ± 1.96SD. IQR and LOAs are presented in brackets (IQR/LOAs).

Parameter	Standing	Seated	Supine	Side R	Side L
Normalized amplitude	6.8% (21.5/±12.2)	9.0% (26.0/±18.0)	0.8% (33.2/±33.7)	4.4% (28.7/±21.1)	1.1% (27.0/±20.4)
Respiratory rate (bpm)	0% (3.5/±5.9)	0% (8.9/±7.9)	0% (15.0/±10.6)	0% (6.8/±8.6)	0% (4.7/±6.3)
Normalized minute ventilation	5.9% (23.4/±12.5)	7.0% (27.6/±16.2)	3.4% (34.9/±38.0)	3.6% (29.9/±22.6)	1.2% (28.1/±20.7)
Duty cycle (%)	−0.29 (0.18/±0.07)	−0.31 (0.20/±0.08)	−0.32 (0.21/±0.12)	−0.30 (0.18/±0.05)	−0.28 (0.18/±0.06)

**Table 3:** Exercise test and recovery, all intensities, all parameters. Aggregated data are expressed as relative error median percentage, interquartile range (IQR) and limits of agreement (LOAs), computed as ±1.96SD. IQR and LOAs are presented in brackets (IQR/LOAs).

Parameter	Low Intensity	Medium Intensity	High Intensity	Maximum Intensity	Recovery Phase 1	Recovery Phase 2
Normalized amplitude	−11.1% (24.9/±56.5)	−29.6% (18.5/±131.5)	−36.4% (14.8/±194.0)	−39.7% (13.8/±188.1)	−34.3% (14.5/±189.1)	−24.8% (20.6/±128.0)
Respiratory rate (bpm)	0% (22.4/±25.8)	0% (18.9/±19.3)	0% (17.1/±19.6)	0% (15.9/±31.5)	0% (16.2/±30.8)	0% (20.0/±50.8)
Normalized minute ventilation	−9.3% (28.4/±57.1)	−33.3% (18.1/±160.6)	−36.0% (13.6/±296.0)	−40.6% (14.1/±373.7)	−33.7% (14.7/±307.0)	−22.8% (22.1/±168.1)
Duty cycle (%)	−0.21 (0.26/±0.10)	−0.27 (0.24/±0.11)	−0.24 (0.25/±0.13)	−0.23 (0.33/±0.13)	−0.14 (0.18/±0.10)	−0.17 (0.22/±0.09)

**Table 4:** p-values of the tests at rest (all different positions) and of the exercise tests (all different intensities) obtained from the One Way Kruskal–Wallis test.

Parameter	*p*-Value (Test at Rest)	(*p*-Value Exercise Test)
Normalized amplitude	0.107	<0.01
Normalized minute ventilation	0.174	0.001
Respiratory rate (bpm)	0.932	0.631
Duty cycle	<0.05	<0.01

## References

[1] Fieselmann J.F., Hendryx M.S., Helms C.M., Wakefield D.S. (1993). Respiratory rate predicts cardiopulmonary arrest for internal medicine inpatients. J. Gen. Intern. Med..

[2] Cretikos M.A., Bellomo R., Hillman K., Chen J., Finfer S., Flabouris A. (2008). Respiratory rate: The neglected vital sign. Med. J. Aust..

[3] Yañez A.M., Guerrero D., Calle-Rubio M., Torrent M., Ussetti P., Alvarez-Sala J.L., de Molina R.M., Falcones M.V., Sauleda J., Gay M.F. (2012). Monitoring breathing rate at home allows early identification of COPD exacerbations. Chest.

[4] Shah S.A., Velardo C., Farmer A., Tarassenko L. (2017). Exacerbations in Chronic Obstructive Pulmonary Disease: Identification and Prediction Using a Digital Health System. J. Med. Internet Res..

[5] Chiarugi F., Karatzanis I., Zacharioudakis G., Meriggi P., Rizzo F., Stratakis M., Louloudakis S., Biniaris C., Valentini M., Di Rienzo M. Measurement of heart rate and respiratory rate using a textile-based wearable device in heart failure patients. Proceedings of the 2008 Computers in Cardiology.

[6] Sun Q., Qiu H., Huang M., Yang Y. (2020). Lower mortality of COVID-19 by early recognition and intervention: Experience from Jiangsu Province. Ann. Intensive Care.

[7] Al Rajeh A., Hurst J. (2016). Monitoring of Physiological Parameters to Predict Exacerbations of Chronic Obstructive Pulmonary Disease (COPD): A Systematic Review. J. Clin. Med..

[8] Massaroni C., Nicolò A., Schena E., Sacchetti M. (2020). Remote Respiratory Monitoring in the Time of COVID-19. Front. Physiol..

[9] Villar R., Beltrame T., Hughson R.L. (2015). Validation of the Hexoskin wearable vest during lying, sitting, standing, and walking activities. Appl. Physiol. Nutr. Metab..

[10] Sarmento A., Vignati C., Paolillo S., Lombardi C., Scoccia A., Nicoli F., Mapelli M., Leonardi A., Ossola D., Rigoni R. (2018). Qualitative and quantitative evaluation of a new wearable device for ECG and respiratory Holter monitoring. Int. J. Cardiol..

[11] Chu M., Nguyen T., Pandey V., Zhou Y., Pham H.N., Bar-Yoseph R., Jain R., Radom-Aizik S., Cooper D.M., Khine M. (2019). Respiration rate and volume measurements using wearable strain sensors. NPJ Digit. Med..

[12] Naranjo-Hernández D., Talaminos-Barroso A., Reina-Tosina J., Roa L.M., Barbarov-Rostan G., Cejudo-Ramos P., Marquez-Martin E., Ortega-Ruiz F. (2018). Smart vest for respiratory rate monitoring of copd patients based on non-contact capacitive sensing. Sensors.

[13] Cesareo A., Previtali Y., Biffi E., Aliverti A. (2019). Assessment of breathing parameters using an inertial measurement unit (IMU)-based system. Sensors.

[14] Liu G.Z., Guo Y.W., Zhu Q.S., Huang B.Y., Wang L. (2011). Estimation of respiration rate from three-dimensional acceleration data based on body sensor network. Telemed. J. E-Health.

[15] Massaroni C., Venanzi C., Silvatti A.P., Presti D.L., Saccomandi P., Formica D., Giurazza F., Caponero M.A., Schena E. (2018). Smart textile for respiratory monitoring and thoraco-abdominal motion pattern evaluation. J. Biophotonics.

[16] Downey C., Ng S., Jayne D., Wong D. (2019). Reliability of a wearable wireless patch for continuous remote monitoring of vital signs in patients recovering from major surgery: A clinical validation study from the TRaCINg trial. BMJ Open.

[17] Whitlock J., Sill J., Jain S. (2020). A-spiro: Towards continuous respiration monitoring. Smart Health.

[18] Liang Q., Xu L., Bao N., Qi L., Shi J., Yang Y., Yao Y. (2019). Research on non-contact monitoring system for human physiological signal and body movement. Biosensors.

[19] Aliverti A. (2017). Wearable technology: Role in respiratory health and disease. Breathe.

[20] Angelucci A., Aliverti A. (2020). Telemonitoring systems for respiratory patients: Technological aspects. Pulmonology.

[21] Nicolò A., Massaroni C., Passfield L. (2017). Respiratory Frequency during Exercise: The Neglected Physiological Measure. Front. Physiol..

[22] Antonelli A., Stanzi A., Mazza F., Venturino M., Noceti P., Guilizzoni D., Aliverti A., Melloni G. (2018). Validation study of the Airgo™ device for continuous monitoring of respiratory function. Eur. Respir. J..

[23] Qi W., Aliverti A. (2019). A Multimodal Wearable System for Continuous and Real-time Breathing Pattern Monitoring During Daily Activity. IEEE J. Biomed. Health Inform..

[24] Romei M., Mauro A.L., D’Angelo M.G., Turconi A.C., Bresolin N., Pedotti A., Aliverti A. (2010). Effects of gender and posture on thoraco-abdominal kinematics during quiet breathing in healthy adults. Respir. Physiol. Neurobiol..

